# Polymorphism in *dhfr/dhps* genes, parasite density and *ex vivo* response to pyrimethamine in *Plasmodium falciparum* malaria parasites in Thies, Senegal^☆^

**DOI:** 10.1016/j.ijpddr.2013.07.001

**Published:** 2013-08-12

**Authors:** Daouda Ndiaye, Baba Dieye, Yaye D. Ndiaye, Daria Van Tyne, Rachel Daniels, Amy K. Bei, Aminata Mbaye, Clarissa Valim, Amanda Lukens, Souleymane Mboup, Omar Ndir, Dyann F. Wirth, Sarah Volkman

**Affiliations:** a Faculty of Medicine and Pharmacy, University Cheikh Anta Diop, Dakar, PO Box 5005, Dakar, Senegal; b Department of Immunology and Infectious Diseases, Harvard School of Public Health, Boston, MA 02115, USA

**Keywords:** *Plasmodium falciparum*, *dhfr/dhps*, Pyrimethamine, Resistance

## Abstract

Resistance to sulfadoxine–pyrimethamine (SP) in *Plasmodium falciparum* malaria parasites is associated with mutations in the dihydrofolate reductase (*dhfr*) and dihydropteroate synthase (*dhps*) genes, and these mutations have spread resistance worldwide. SP, used for several years in Senegal, has been recommended for intermittent preventive treatment for malaria in pregnancy (IPTp) and has been widely implemented since 2003 in this country. There is currently limited data on SP resistance from molecular marker genotyping, and no data on pyrimethamine *ex vivo* sensitivity in Senegal. Molecular markers of SP resistance and pyrimethamine *ex vivo* sensitivity were investigated in 416 parasite samples collected from the general population, from the Thies region between 2003 and 2011. The prevalence of the N51I/C59R/S108N triple mutation in *dhfr* increased from 40% in 2003 to 93% in 2011. Furthermore, the prevalence of the *dhfr* N51I/C59R/S108N and *dhps* A437G quadruple mutation increased, from 20% to 66% over the same time frame, then down to 44% by 2011. There was a significant increase in the prevalence of the *dhfr* triple mutation, as well as an association between *dhfr* genotypes and pyrimethamine response. Conversely, *dhps* mutations in codons 436 and 437 did not show consistent variation between 2003 and 2011. These findings suggest that regular screening for molecular markers of antifolate resistance and *ex vivo* drug response monitoring should be incorporated with ongoing *in vivo* efficacy monitoring in areas where IPTp-SP is implemented and where pyrimethamine and sulfa drugs are still widely administered in the general population.

## Highlights

• Prevalence of *dhfr/dhps* mutations increased significantly between 2003 and 2011.
• Triple mutant *dhfr* 51I/59R/108N increased, from 40% in 2003 to 93% in 2011.
• Quadruple mutant *dhfr* and *dhps* 437G increased, from 20% to 66% then down.
• A strong correlation between *ex vivo* response to pyrimethamine and *dhfr* genotype.

## Graphical abstract

**Figure:**
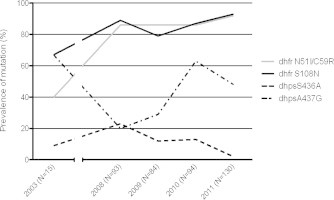

## 1 Introduction

*Plasmodium falciparum* malaria continues to be a major global cause of mortality and morbidity. Malaria treatment and control has been complicated by the emergence of resistance to widespread antimalarial drug use. The most common method for measuring antimalarial resistance is estimating the *in vivo* efficacy of the antimalarial, such as sulfadoxine–pyrimethamine (SP). Since 2003, SP has been used in the intermittent preventive treatment for pregnant women (IPTp-SP) in many Sub-Saharan African countries, including in Senegal since 2003 (WHO, 2004). sulfadoxine–pyrimethamine in combination with amodiaquine was also recently recommended by the WHO for seasonal malaria chemoprevention (SMC) in some malaria-endemic countries (WHO Global Malaria Programme, 2012). Due to the recent recommendation to use artemisinin combination therapies (ACTs) for the treatment of uncomplicated malaria (WHO, 2010), it is no longer acceptable to carry out *in vivo* efficacy studies of SP used alone for the treatment of uncomplicated malaria. Nonetheless, it is critical to assess parasite SP resistance in order to monitor the efficacy of SP use in IPTp and SMC.

Antimalarial drug sensitivity testing provides information on the frequency of resistant phenotypes among the populations of parasites being transmitted, as well as the possible cross-resistance patterns of antimalarial drugs. Isolates are defined as resistant to pyrimethamine when the 50-percent inhibitory concentration (IC_50_) is greater than 2000 nM (Aubouy et al., 2003). *In vitro* methods to measure parasite resistance to individual components is a useful adjunct to *in vivo* studies (Desjardins et al., 1979; Smilkstein et al., 2004; Baniecki et al., 2007; Laufer et al., 2007; Kurth et al., 2009; Ndiaye et al., 2010). *In vivo* and *in vitro* drug sensitivity tests present numerous technical and cost limitations, and these limitations have led to a search for genetic markers of resistance.

As *in vivo* drug efficacy cannot be routinely monitored in IPTp-SP, an alternative method to track SP resistance is to study the frequency of molecular markers that are associated with SP resistance in the parasite population. The mechanism of action of SP is well documented: point mutations at codons 50, 51, 59, 108, and 164 in the *dhfr* gene (Bzik et al., 1987; Cowman et al., 1988; Peterson et al., 1988, 1990; Foote et al., 1990; Basco et al., 1995; Reeder et al., 1996) are found to confer resistance to pyrimethamine, while mutations at codons 437, 540, 581, and 613 of the *dhps* gene confer resistance to sulfadoxine (Brooks et al., 1994; Triglia and Cowman, 1994; Bickii et al., 1998; Warhurst, 2001; Warsame et al., 2001). The single *dhfr* 108 mutation can increase *in vitro* resistance to pyrimethamine by 100-fold relative to wild-type (Reeder et al., 1996; Sirawaraporn et al., 1997), and the progressive addition of mutations altering Cys50 to Arg (C50R), Asn51 to Ile (N51I), Cys59 to Arg (C59R), and Ile164 to Leu (I164L) in the gene can yield higher levels of SP resistance both *in vitro* and *in vivo* (Reeder et al., 1996; Sirawaraporn et al., 1997). The triple *dhfr* mutant genotype consisting of N51I, C59R, and S108N shows *in vitro* resistance to pyrimethamine that is 225 times higher than a wild-type lab strain (Basco et al., 1995; Nzila-Mounda et al., 1998), and has a strong association with *in vivo* SP treatment failure (Basco et al., 1998; Kublin et al., 2002; Happi et al., 2005). Sulfadoxine is the most common of the sulfones and sulfonamide class of drugs used in prophylaxis and/or treatment for human malaria caused by *P. falciparum*. A change at codon A437G in *dhps* is the first step in resistance to sulfa drugs, followed by sequential mutations at K540E, A581G, and A613S/T, which cause a further increase in drug resistance (Triglia et al., 1997). The quintuple mutant genotype consisting of the double *dhps* mutant genotype (A437G, K540E) in combination with the *dhfr* triple mutant genotype (S108N, N51I, C59R) also predicts clinical failure (Omar et al., 2001; Kublin et al., 2002; Mugittu et al., 2004; Staedke et al., 2004; Alker et al., 2008).

In Senegal, malaria remains a formidable public health issue, causing significant morbidity and mortality in infants and pregnant women (WHO Malaria Report, 2012). In the absence of an effective vaccine, the National Malaria Control Program has followed WHO recommendations for IPTp-SP since 2003. The rapid spread of SP-resistant parasites highlights the need for regular monitoring of *ex vivo* parasite sensitivity to pyrimethamine and *dhfr/dhps* mutations in countries like Senegal, where SP has been widely used for several years.

## 2 Materials and methods

### 2.1 Study population

This study was conducted from 2003 to 2011 at the Service de Lutte Anti-Parasitaire (SLAP) clinic, in the Thies region of Senegal. Thies is an urban area, 70 km from the capital city of Dakar, where malaria is hypoendemic (1 < EIR < 5) (Trape et al., 1992; Faye et al., 1995; Thomas et al., 2002). Individuals seeking treatment for uncomplicated *P. falciparum* malaria at the SLAP clinic in Thies were tested for malaria infection by microscopy and rapid diagnostic test (RDT). Patients that presented with symptoms consistent with mild malaria, including fever and a positive blood slide with only *P. falciparum*, were offered enrollment into the study. Exclusion criteria included severe disease and/or history of recent treatment with anti-malarial drugs. The Human Subjects Committee of Harvard School of Public Health in Boston, (protocol #P10256-127) and the Ethics Committee of the Senegal Ministry of Health in Dakar (Protocol #16330) both approved the protocols used in these studies.

### 2.2 Blood sample collection

For screening, thin and thick blood films were performed for parasite detection and identification of malaria parasite species and parasite counts by light microscopy using Giemsa stain (10% dilution). Blood samples were collected either on Whatman FTA filter papers (Whatman catalog #WB120205) or via venous blood draw from consenting patients, who were then treated with the first line treatment regimen according to the guidelines of the Senegalese Ministry of Health. Filter papers alone were collected in 2003 for genotyping, while both filter papers for genotyping and venous blood for the *ex vivo* drug assay were collected from 2008–2011. Blood samples were collected after written informed consent was obtained from each patient or their parent/guardian.

### 2.3 DNA extraction and genotyping

DNA extraction was performed from filter paper blood spots using a QIAamp DNA Minikit (Qiagen #51183) following manufacturer’s instructions (Thomas et al., 2002). In 2003, *dhfr* and *dhps* mutations were analyzed using the PCR-RFLP protocol (Ndiaye et al., 2005), with primers used to amplify the region that includes codons 50, 51, 59, 108, and 164 in *dhfr* and codons 436, 437, 540, 581, and 613 in *dhps*. In 2008, 2009, 2010, and 2011, haplotypes were analyzed using High Resolution Melting genotyping (Daniels et al., 2012) (Table 1).

### 2.4 Ex vivo drug assay

Parasites were drug tested using the *ex vivo* DAPI assay (Ndiaye et al., 2010). Briefly, 180 μL of media plus parasitized erythrocytes at 2% hematocrit and parasitemia between 0.4% and 1% were distributed into 96-well plates preloaded with 20 μL of serially diluted pyrimethamine, prepared in duplicate wells. Pyrimethamine was obtained from Sigma (catalog #P7771) and stock solutions were prepared in DMSO. Drug plates were prepared in a single batch and frozen prior to use, and lab strain controls (3D7 and Dd2) were used to validate each plate batch. Two sets of serial dilutions were prepared in unsupplemented RPMI and distributed in duplicate into 96 well black plates: a series with high pyrimethamine concentrations from 295 to 2.7 μM, followed by a series of low pyrimethamine concentrations from 1366.6 to 3.3 nM. Samples that had parasitemia greater than 1% were diluted with leukocyte-free O+ donor red blood cells resulting in a final parasitemia of 0.4–1%. Parasites were cultured for 48–72 h at 37° Celsius in standard gas conditions (1% O_2_, 5% CO_2_, and 94% N_2_) before addition of 4′,6-diamidino-2-phenylindole (DAPI) solution, as previously described (Ndiaye et al., 2010). After culture, drug plates were read using a fluorescent plate reader. The 50% inhibitory concentration (IC_50_) was calculated using GraphPad Prism v5.0, estimated by non-linear regression analysis of log_10_-transformed dose-response curves.

### 2.5 Statistical analysis

Two-tailed Fisher’s exact test was used to determine whether mutant allele frequencies increased by year (2003 versus 2011). Mann–Whitney *U* test was used to determine whether median IC_50_ values differed for parasites with wild-type and mutant alleles. GraphPad Prism was used to analyze IC_50_s for pyrimethamine. For all statistical tests, alpha = 0.05.

## 3 Results

### 3.1 Patient ages and parasite densities

We monitored 416 Senegalese patients from 2003–2011 with ages ranging from 2 to 65 years. Patient parasitemia increased between 2003 and 2008 (*p* < 0.002) (Table 2).

### 3.2 Prevalence of *dhfr* and *dhps* point mutations

**Fig. 1:**
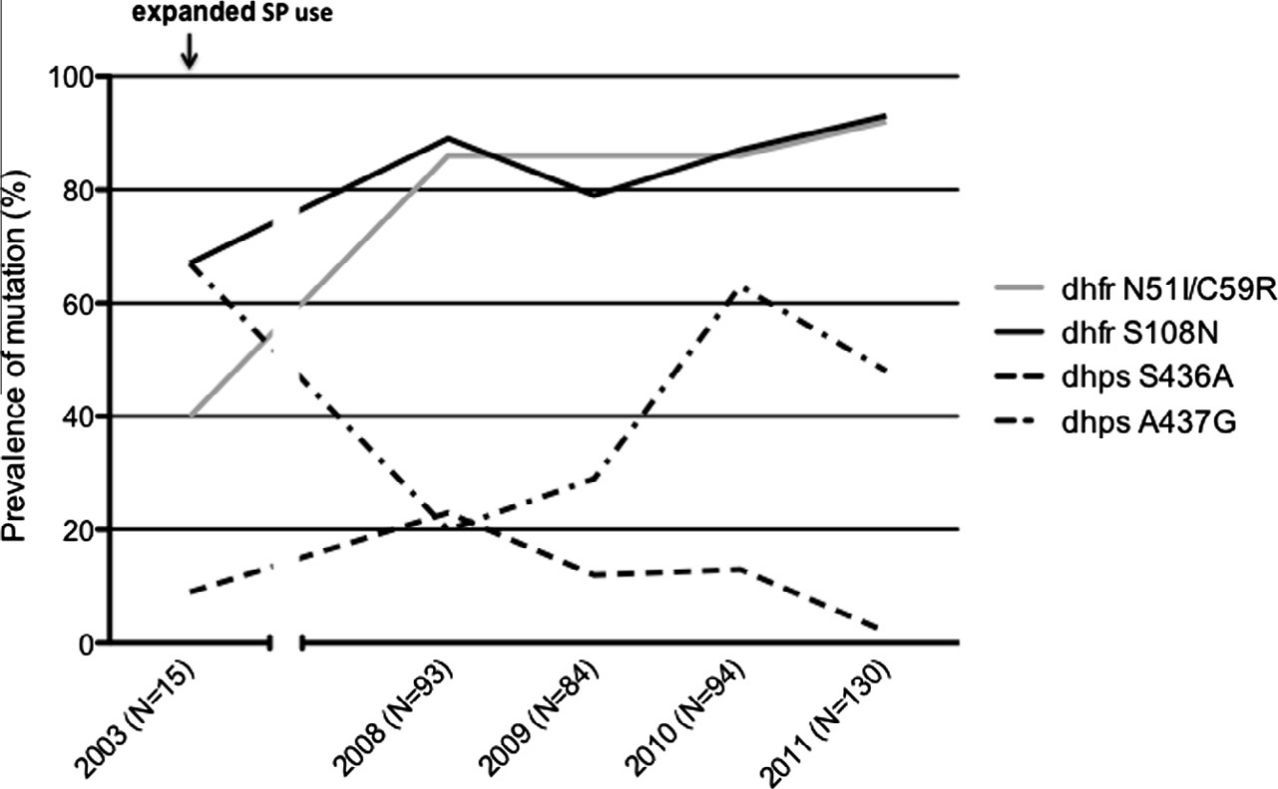
Evolution of *dhfr* N51I/C59R/S108N and *dhps* mutation prevalence after expanded SP use in Thies, Senegal. The prevalence of the *dhfr* mutant alleles for both 51I/59R and 108N increased significantly between 2003 and 2011 (Fischer’s exact, *p* = 0.0002). *Dhps* mutation individually fluctuated (no significant change) between 2003 and 2011 (Ndiaye et al., 2005), (Daniels et al., 2012).

A total of 416 *P. falciparum* samples collected between 2003 and 2011 were successfully genotyped for the following mutations: *dhfr* C50R, N51I, C59R, S108N, and I164L, and *dhps* S436A, A437G, K540E, A581G, and A613S/T. We did not detect the following mutations: *dhfr* C50R and I164L, and *dhps* K540E, A581G, and A613S/T. Fig. 1 shows that the prevalence of mutations in *dhfr* in 2003 was between 40% (N51I and C59R) and 67% (S108N), and rose to 93% or greater in 2011, resulting in a significant increase (Fischer’s exact, *p* = 0.0002) from 2003 to 2011. *Dhps* mutations individually fluctuated (no significant change) between 2003 and 2011. The *dhps* mutations at codons 436 and 437 did not show significant variation between 2003 and 2011 (*p* = 0.08), but rather fluctuated between 2% and 23% (S436A) and between 20% and 67% (A437G). Among all isolates, no more than 6 isolates had mixed alleles at any given *dhfr* or *dhps* locus.

**Fig. 2:**
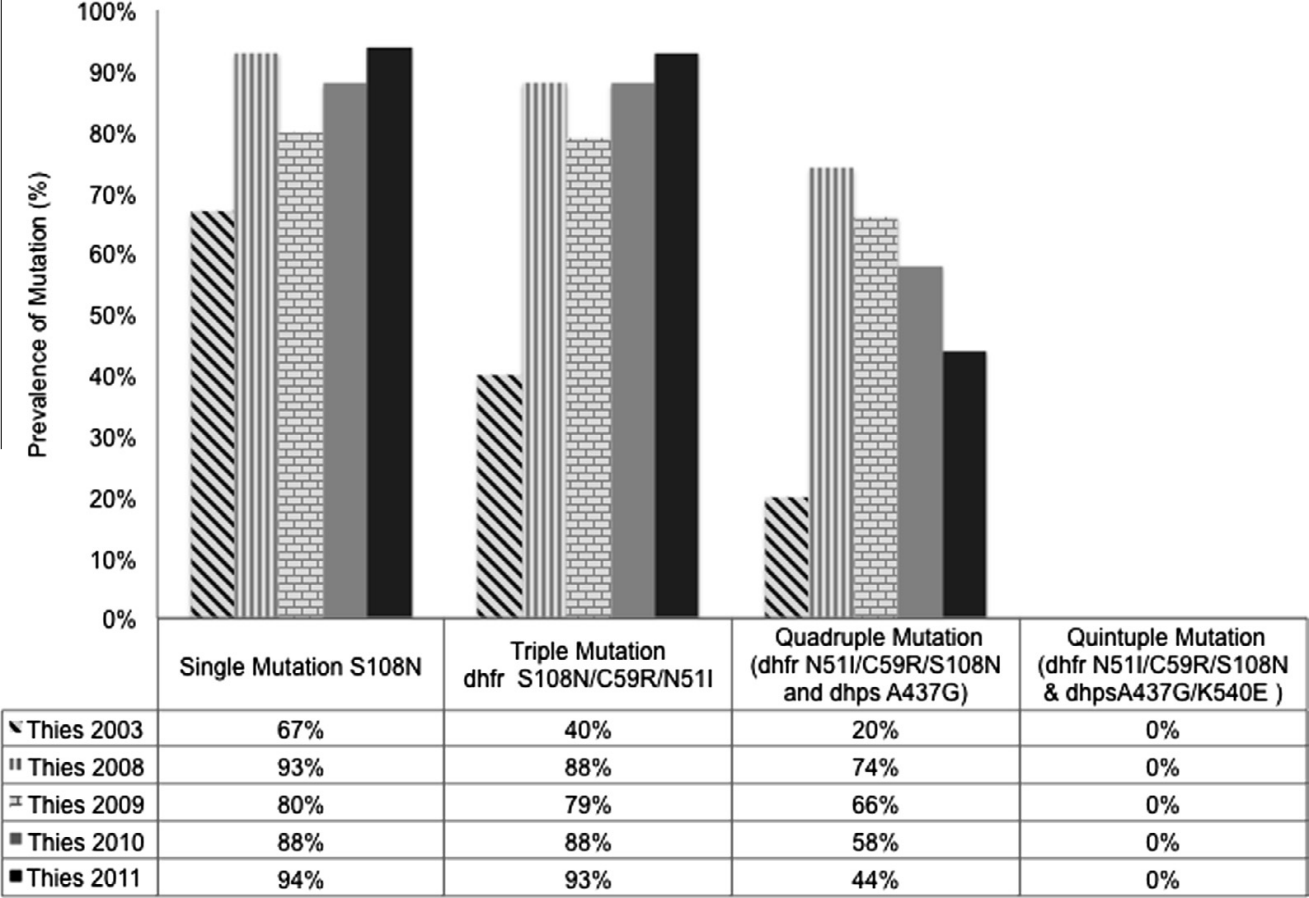
Haplotype frequencies and number of mutations in *dhfr* codons 51, 59, 108 and *dhps* 436 and 437, present in *P. falciparum* isolates from Thies between 2003 and 2011. Haplotype frequencies were determined by HRM (in 2003) or PCR-RFLP (in 2008–2011). Significant increases were detected using Fisher’s exact test to detect differences between 2003 and 2011. The prevalence of the *dhfr* 51I/59R/108N triple mutant genotype increased from 40% in 2003 to 93% in 2011 (Fischer’s exact, *p* = 0.0002); and the prevalence of the *dhfr* 51I/59R/108N and *dhps* 437G quadruple mutant genotype increased from 20% to 44% over the same time period. 2003 data was previously reported in Ndiaye et al. (2005).

We observed that the prevalence of the *dhfr* 51I/59R/108N triple mutant genotype increased significantly from 40% in 2003 to 93% in 2011 (Fisher’s exact, *p* = 0.0002); and the prevalence of the *dhfr* 51I/59R/108N and *dhps* 437G quadruple mutant genotype also increased from 20% to 44% over the same time period (Fig. 2). We did not observed the appearance of the *dhfr* 51I/59R/108N and *dhps* 437G/540E quintuple mutant genotype. The quadruple mutant genotype increased between 2003 and 2008, and decreased between 2008 and 2011.

### 3.3 *Ex vivo* susceptibility of Senegalese *P. falciparum* isolates to pyrimethamine

A DAPI-based *ex vivo* assay was used to test pyrimethamine sensitivity in 66 parasite isolates from 2011 (Ndiaye et al., 2010). 3D7 and Dd2 parasites were used as control strains; their IC_50_s were 47.2 and 49,464 nM, respectively. Out of the 66 isolates, 56 (84.8%) were found to be resistant to pyrimethamine with IC_50_s greater than 2000 nM. The median pyrimethamine IC_50_ was 25,125 nM with a minimum of 2.4 nM and a maximum of 11,107 nM (Table 3). The median IC_50_ among sensitive isolates and resistant isolates were 247.8 (2.4–1503) nM, and 30,705 (2259–201,046) nM, respectively (Table 4).

### 3.4 Correlation between *dhfr* polymorphisms and pyrimethamine *ex vivo* susceptibility

**Fig. 3:**
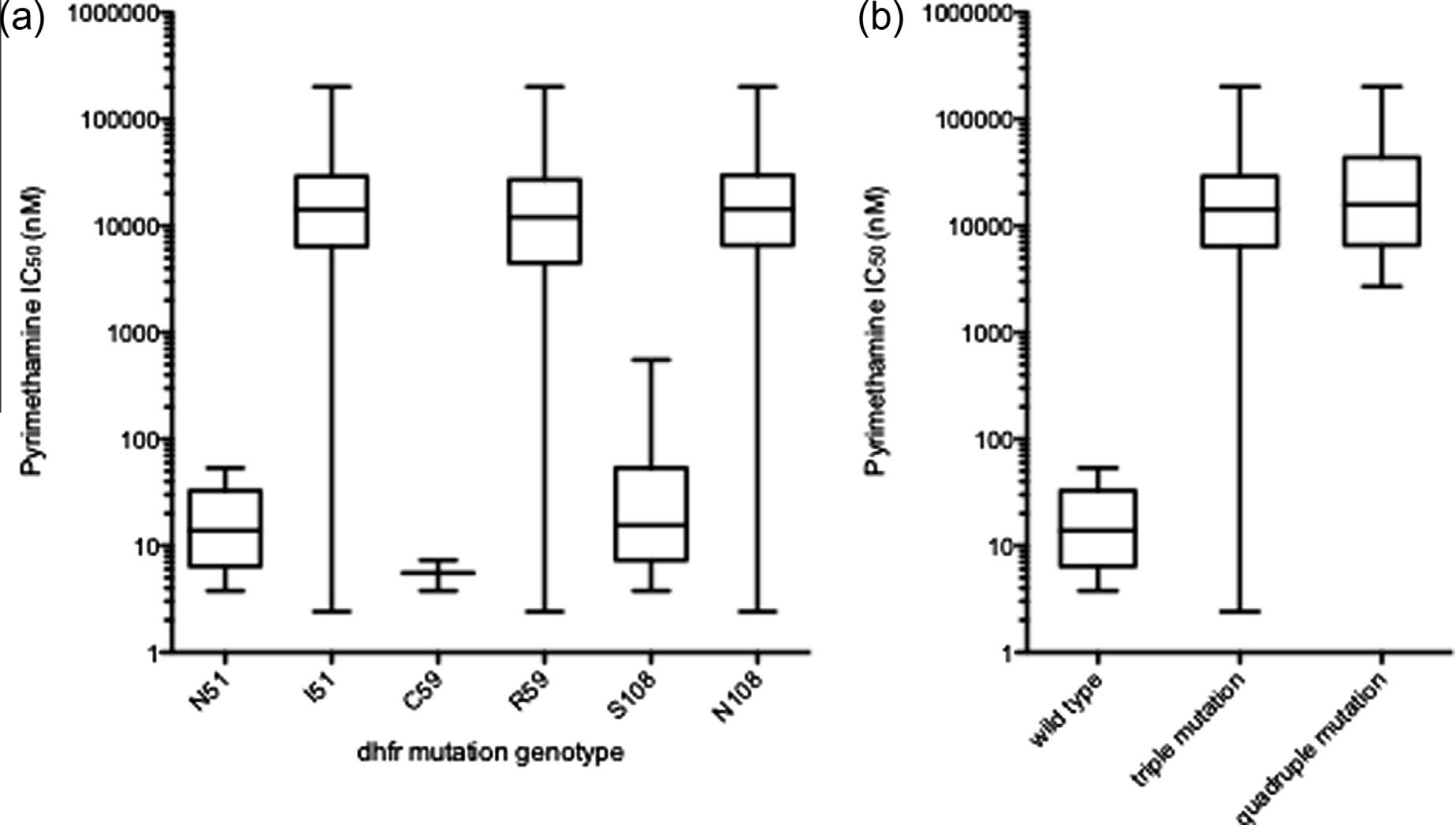
IC_50_ (nM) comparisons between mutant and wild type alleles at codons 51, 59, and 108 in *dhfr* and comparison between parasites with wild type versus *dhfr* N51I/C59R/S108N triple mutation and *dhfr* N51I/C59R/S108N and *dhps* A437G quadruple mutation. (a) We found significant increases in the geometric mean IC_50_ values for *ex vivo* pyrimethamine susceptibility between mutant and wild type alleles (Mann–Whitney *U* test, *p* = 0.0001). Pyrimethamine IC_50_s were measured *ex vivo* in 2011 using the DAPI drug assay. (b) IC_50_s were different between wild type and *dhfr* N51I/C59R/S108N triple mutation parasites, as well as between wild type and *dhfr* N51I/C59R/S108N and *dhps* A437G quadruple mutation parasites (Mann–Whitney *U* test, *p* = 0.0002 for both comparisons).

The correlation between the *dhfr* mutation and resistance to pyrimethamine measured *ex vivo* was verified by our study. We found significant increases in the geometric means of the IC_50_ values for *ex vivo* pyrimethamine susceptibility among parasites bearing single mutations within *dhfr* (Fig. 3, Mann–Whitney *U* test, *p* = 0.0001). In the pyrimethamine resistant isolates, the mean IC_50_ for parasites with the mutant 108N allele (*N* = 58, mean IC_50_ = 31,181 nM, CI95% 30,002–32,606) was 324 times higher than the mean IC_50_ for parasites with the wild-type S108 allele (*N* = 9, mean IC_50_ = 96 nM, CI95% 94.7–97). The mean IC_50_ for parasites with the mutant 51I allele (*N* = 56, mean IC_50_ = 31,540 nM, CI95% 30,160–32,100) was 240 times higher than the mean IC_50_ for parasites with the wild-type N51 allele (*N* = 6, mean IC_50_ = 131 nM, CI95% 130.7–133.6). Likewise, the mean IC_50_ for parasites with the mutant 59R allele (*N* = 57, mean IC_50_ = 31,021 nM, CI95% 30,259–32,119) was 5640 times higher than the mean IC_50_ for parasites with the wild-type C59 allele (*N* = 2, mean IC_50_ = 5.5 nM, CI95% 5.2–5.8) (Fig. 3a). We observed a mean IC_50_ 1000 times higher between wild type and triple mutant *dhfr* 51I/59R/108N parasites, (Mann–Whitney *U* test, *p* = 0.0002), as well as between wild type and *dhfr* 51I/59R/108N and *dhps* 437G quadruple mutant parasites (Mann–Whitney *U* test, *p* = 0.0002) (Fig. 3b). Four of the 10 isolates that were sensitive to pyrimethamine also had the mutation at *dhfr* 108, while all six parasites with the *dhfr* S108 wild-type allele were sensitive to pyrimethamine. The six isolates with the wild-type allele at *dhfr* C51 were also sensitive to pyrimethamine, and the same observation was made for isolates with the wild-type *dhfr* C59 allele. This illustrates that all parasites with the wild-type alleles at *dhfr* 51, 59 and 108 were sensitive to pyrimethamine, and the correlation between *dhfr* mutation and phenotypes was statistically significant (Fisher exact, *p* = 0.00001).

## 4 Discussion

In 2003, Senegal adopted intermittent preventive treatment for pregnant women (IPTp) using sulfadoxine–pyrimethamine (SP). At the same time, between 2003 and 2004, Senegal switched to sulfadoxine–pyrimethamine with amodiaquine as the first-line therapy for uncomplicated malaria in response to increasing chloroquine resistance (WHO Roll Back Malaria Focus on Senegal, 2010). In 2005, Senegal adopted artemisinin combination therapies (ACTs) as first line treatment for uncomplicated malaria. The results reported here were obtained from samples collected from the general population in an urban site with expanded SP use, and this is one of the few reports that includes both *dhfr/dhps* polymorphisms and *ex vivo* drug phenotype data. Previous studies carried out in Senegal and other West African countries have focused on rural sites and studied *P. falciparum* polymorphisms without assessment of corresponding *ex vivo* phenotypes.

Our results show an increase in the prevalence of parasites bearing individual *dhfr* mutations at codons 51, 59, and 108 in an interval of eight years. Furthermore, the number of parasites with all three *dhfr* mutations increased from 40% in 2003 (Ndiaye et al., 2005), to 93% in 2011. Emergence of the *dhfr* 51I/59R/108N triple mutant has been observed in countries using sulfadoxine–pyrimethamine alone or in combination, as first line treatment for uncomplicated malaria as reported in Africa and elsewhere (Bwijo et al., 2003; Griffin et al., 2010; Malisa et al., 2010; Raman et al., 2010; Yusuf et al., 2010; Zakeri et al., 2010; Mula et al., 2011; Mombo-Ngoma et al., 2011; Naidoo and Ropper, 2011). A similar increase in the *dhfr* N51I/C59R/S108N triple mutation has been observed after IPT in children in southern Senegal (Faye et al., 2011), as well as in rural regions in Mali (Dicko et al., 2010) and southern Mozambique (Enosse et al., 2008), with *dhfr* mutations being an important predictive risk factor of *in vivo* resistance (Boumbou-Moukoko et al., 2009; Picot et al., 2009). *Dhps* mutations individually fluctuated (no significant change) between 2003 and 2011 in this study, but when considered in combination with *dhfr* mutations, the number of parasites with an additional mutation at *dhps* 437 (*dhfr* N51I/C59R/S108N and *dhps* A437G quadruple mutation) increased from 2003 to 2008 and then steadily decreased until 2011. Interestingly, we found that mutations at *dhps* codons 436 and 437 were not always inherited together, despite residing very close to each other on the chromosome (Bwijo et al., 2003; Bouyou-Akotet et al., 2010).

The quintuple mutant *dhfr* 51I/59R/108N and *dhps* 437G/ 540E has not been previously observed in Senegal (Ndiaye et al., 2005, 2006; Henry et al., 2006; Faye et al., 2011), or in Mali (Dicko et al., 2010). The *dhfr* I164L mutation was also not found in this study. The combination of *dhfr* C59R and *dhps* K540E mutations, which predict clinical failure of sulfadoxine–pyrimethamine (Basco et al., 2000; Kublin et al., 2002; Talisuna et al., 2004; McCollum et al., 2012), were also not found in our study.

We found a correlation between the *dhfr* S108N single mutation and pyrimethamine resistance, and a correlation between the *dhfr* N51I/C59R/S108N triple mutation, as well as the *dhfr* N51I/C59R/S108N and *dhps* A437G quadruple mutation, and pyrimethamine resistance. Overall, we found a significant difference in the geometric mean IC_50_ values for pyrimethamine (*p* = 0.0009) between parasites possessing wild-type and resistant alleles in *dhfr*, as has been reported by others (Andriantsoanirina et al., 2011). We confirmed the existence of an association between the *dhfr* genotype and chemosensitivity to pyrimethamine in *P. falciparum* isolates from Thies, as the increase in the number of mutations was associated with an increase in *ex vivo* resistance to pyrimethamine, similar to what has been observed in Gabon (Aubouy et al., 2003), Central African Republic (Menard et al., 2006), and Cote D’Ivoire (Djaman et al., 2007). However, some parasites harbored the N51I, C59R, and S108N mutations in *dhfr* but were still susceptible to pyrimethamine as reported in isolates from Brazil (Petersen et al., 1991), and Gabon (Aubouy et al., 2003) for the *dhfr* S108N mutation and Papua New Guinea (Reeder et al., 1996) for *dhfr* S108N and *dhfr* C59R. Further sequencing of these parasites for possible compensatory mutations may explain this finding. The *ex vivo* assay data does not permit strong conclusions because we obtained *ex vivo* pyrimethamine data from only 1 year; however, the high rates of pyrimethamine *ex vivo* resistance in this study are correlated with high rates of the *dhfr* N51I/C59R/S108N triple mutation.

The use of SP in IPTp may not be the only driver of parasite polymorphisms in this population, because Senegal has used sulfadoxine and/or pyrimethamine in the national antimalarial treatment plan for many years, and furthermore these drugs are still being used in antibacterial combination therapy. Nonetheless, this type of general population survey could form part of the monitoring system for IPTp as an alert strategy plan, because the genetic and phenotypic diversity among parasites infecting the general population in very low transmission areas like Thies (EIR < 5), likely reflect the parasites circulating among pregnant women.

Our study is not without limitations. The small number of patients recruited in 2003 was due to logistical constraints, which were addressed in the following years and allowed for deeper sampling in 2008–2011. The intervening years were also spent developing the DAPI *ex vivo* drug assay (Ndiaye et al., 2010) and High Resolution Melting genotyping (Daniels et al., 2012). The latter technology is a reliable, adaptable, and accessible tool that provides comparable results to PCR-RFLP. Future studies will strengthen the present data set and will provide valuable information for the Senegalese National Malaria Control Program.

In conclusion, our results show an increasing prevalence of *dhfr* N51I/C59R/S108N triple and *dhfr* N51I/C59R/S108N and *dhps* A437G quadruple mutations between 2003 and 2011 in Thies, Senegal. This study suggests that intermittent drug pressure with SP is selecting parasites with mutant alleles. The use of SP is not only implemented in IPTp, but also recently for seasonal malaria chemoprevention in children, thus surveillance of molecular markers of drug resistance and *ex vivo* drug sensitivity assays should be an integral part of planned malaria control programs, so that resistance dynamics can be assessed and the most effective treatment can be selected or modified.

## Figures and Tables

**Table 1:** Primer and probe sequences used for PCR-RFLP and high resolution melting assay.

Technique	Gene/SNP ID	Forward primer 5’ > 3’	Reverse primer 5’ > 3’	Probe 5’ > 3’
PCR–RFLP	dhfr.1	ATG GAA CAA GTC TGC GAC GTT TTC	ATG ACA TGT ATC TTT GTC ATC ATT	
	dhfr.2	ATG GAA CAA GTC TGC GAC GTT TTC	ATT GTT ACT AGT ATA TAC ATC GCT	
	dhps.1	CCATTCCTCATGTGTATA CAACAC	CATCTG AAACATCCAATTGTGT GA	
	dhps.2	TATGATTCTTTT TCAGAT GGAGGT	CATCTGAAACATCCAATTGTG TGA	

HRM	N51/C59	ACATTTAGAGGTCTAGGAAATAAAGGAGT	ATATTTACATCTCTTATATTTCAATTTTTC ATATTT TGATTCATTCAC	AAATGTAATTCCCTAGATATGAAATATTTTTGTG CAG-block
	I164	ACAAAGTTGAAGATCTAATAGTTTTACTTGGG	CTGGAAAAAATACATCACATTCATATGTACTATTTATTCTA	AATGTTTTATTATAGGAGGTTCCG-block
	S108	CTGTGGATAATGTAAATGATATGCCTAATTCTA	GACAATATAACATTTATCCTATTGCTTAAAGGT	GGAAGAACAAGCTGGGAAAGCAT-block
	S436/A437	GAATGTTTGAAATGATAAATGAAGGTGCTA	CAGGAAACAGCTATGACGAAATAATTGTAATACAGG TACTACTAAATCTCT	ATCCTCTGGTCCTTTTGTTATACC-block
	K540	GTGTTGATAATGATTTAGTTGATATATTAAATGATATTAGTGC	GTTTATCCATTGTATGTGGATTTCCTCTT	TAATCCAGAAATTATAAAATTATTAAAAAAAAA AAAC-block
	A581	CTTGTATTAAATGGAATACCTCGTTATAGGA	AGTGGATACTCATCATATACATGTATATTTTGTAAG	TTGGATTAGGATTTGCGAAGAAACATGAT CA-block
	A613	CTCTTACAAAATATACATGTATATGATGAGTATCCACTT	CATGTAATTTTTGTTGTGTATTTATTACAACATTTTGA	AAGATTTATTGCCCATTGCATGA-block

**Table 2:** Ages and parasitemias of patients included in this study from 2003 to 2011.

	2003	2008	2009	2010	2011
Number (*n*)	15	93	84	94	130
Median age (years)	17.7 (7–54)	23 (2–55)	23.5 (4–61)	17 (3–65)	16.5 (3–59)

Median parasitemia (lowest–highest) asexual parasite/μL	12,216 (1019–110,000)	18,000 (4500–135,000)	22,500 (2250–351,000)	23,400 (450–585,000)	22,500 (3150–315,000)

**Table 3:** Pyrimethamine IC_50_ values and percentage of resistant parasites tested in 2011.

Drug tested	IC_50_ median (nM) (CI 95%)	Range		Resistant isolates (%) (n/N)
		Min	Max	
Pyrimethamine (*n* = 66)	11,107 (20,104–27,046)	2.4	201,046	84.8% (56/66)

**Table 4:** *Ex vivo* pyrimethamine IC_50_ values for resistant and sensitive isolates.

Isolates tested	Pyrimethamine sensitive isolates (*n* = 10)	Pyrimethamine resistant isolates (*n* = 56)	(Mann–Whitney *U* test) *p*-value
Field isolates (*n* = 66) median (lowest–highest) IC_50_ (nM)	15.5 (2.4–1503)	15,725 (2259–201,046)	0.0001
3D7	47.2	–	
Dd2	–	49,464	
